# Interobserver Variability in the Assessment of Fluorescence Angiography in the Colon

**DOI:** 10.1177/15533506221132681

**Published:** 2022-11-14

**Authors:** Antonio S. Soares, Neil T. Clancy, Sophia Bano, Imran Raza, Michelle Diana, Laurence B. Lovat, Danail Stoyanov, Manish Chand

**Affiliations:** 1 4919 Wellcome EPSRC Centre for Interventional and Surgical Sciences (WEISS), London, UK; 2 Division of Surgery and Interventional Sciences, 4919 University College London, London, UK; 3 Department of Medical Physics and Biomedical Engineering, 4919 University College London, London, UK; 4 Department of Computer Science, 4919 University College London, London, UK; 5 University College London Hospital, 8964 University College London Hospitals NHS Trust, London, UK; 6 IRCAD, Research Institute Against Digestive Cancer, Strasbourg, France; 7 ICube Lab, Photonics for Health, University of Strasbourg, France

**Keywords:** colorectal surgery, image guided surgery, ergonomics and/or human factors study

## Abstract

**Background:**

Fluorescence angiography in colorectal surgery is a technique that may lead to lower anastomotic leak rates. However, the interpretation of the fluorescent signal is not standardised and there is a paucity of data regarding interobserver agreement. The aim of this study is to assess interobserver variability in selection of the transection point during fluorescence angiography before anastomosis.

**Methods:**

An online survey with still images of fluorescence angiography was distributed through colorectal surgery channels containing images from 13 patients where several areas for transection were displayed to be chosen by raters. Agreement was assessed overall and between pre-planned rater cohorts (experts vs non-experts; trainees vs consultants; colorectal specialists vs non colorectal specialists), using Fleiss’ kappa statistic.

**Results:**

101 raters had complete image ratings. No significant difference was found between raters when choosing a point of optimal bowel transection based on fluorescence angiography still images. There was no difference between pre-planned cohorts analysed (experts vs non-experts; trainees vs consultants; colorectal specialists vs non colorectal specialists). Agreement between these cohorts was poor (<.26).

**Conclusion:**

Whilst there is no learning curve for the technical adoption of FA, understanding the fluorescent signal characteristics is key to successful use. We found significant variation exists in interpretation of static fluorescence angiography data. Further efforts should be employed to standardise fluorescence angiography assessment.

## Introduction

Anastomotic leak (AL) in colorectal surgery is still common today^1^ and causes significant patient morbidity^2^ and costs to the health system.^3^ Several strategies have been employed to avoid this complication and minimize its effects, ranging from better patient stratification^4^ to closer monitoring postoperatively (both biochemical^5^ and radiological^6^). Decision making with regard to the anastomotic level and thus perfusion of the bowel conduit is a main determinant in AL. Intraoperatively, the point of proximal transection is based on the extent of disease that is being resected and by assessment of the proximal colon viability. This decision is most commonly based on clinical judgement through the detection of marginal artery pulse, bleeding from the cut edges of the mesentery and tissue colour.

Recently, fluorescence angiography with indocyanine green (ICG) have been used to better assess bowel perfusion and subsequently, choice of transection point. After intravenous injection, ICG binds to albumin in the intravascular compartment and travels through the circulation. It will be present in all tissue that is perfused, and it can be visualised using a specialised camera system incorporating near infrared imaging. The use of fluorescence angiography has been shown to significantly reduce the odds ratio for anastomotic leak in observational studies.^7,8^

However, for FA to be useful it relies on meaningful interpretation of the displayed visual image. There is no documented learning curve for the technical aspects of FA, ie administering the injection, which is understandable but there is also no recommended learning curve for the interpretation of the images. And it is ultimately image interpretation that leads to the clinical decision making. This is often inaccurately referred to as the ‘number of cases used’. Previous work has compared expert assessment with non-experts using a dynamic video assessment tool,^9^ however no consistent cut off was used to define expert. A better understanding of how to standardize the interpretation of ICG images and potentially, build a quantitative model to help in clinical decision making, is a current unmet need.

The aim of this study was to identify user variability in the interpretation of ICG fluorescence angiography and to further determine whether level of seniority and/or experience influenced clinical decision-making when using ICG.

## Methods

### Setting

An online survey, containing 15 pre-selected images showing the proximal colon before anastomosis, was created to assess user interpretation. Users were asked to select the optimal point of transection from a series of options.

### Image selection

**Figure 1.**
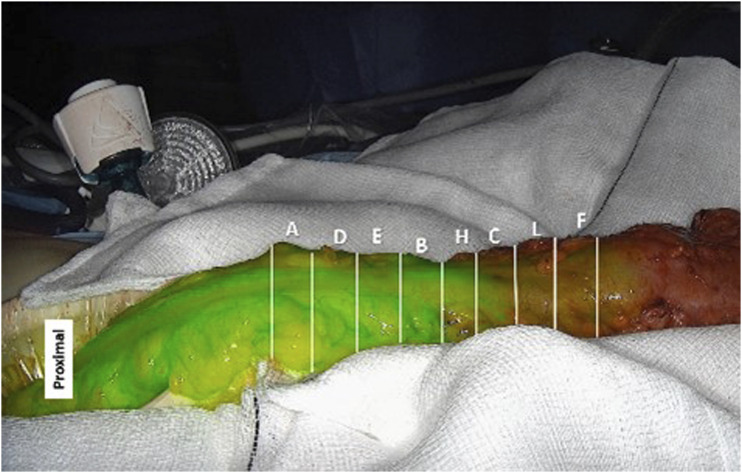
Case #1 image with categories to be selected.

Intraoperative video from a random selection of patients that had undergone surgery and bowel resection using ICG to help in decision making was used. All videos were from a single surgical team at a tertiary referral centre for colorectal surgery. The technique for use of ICG has been extensively described elsewhere.^10^ A still image was selected from the video footage that corresponded to maximal fluorescence intensity for that case as assessed by the operating surgeon. For each image, the length of colon was divided into equal segments representing the transection options as shown in Figure 1 (full survey shown in supplementary figure 1). These were manually segmented along the longitudinal axis of the colon with an approximate distance of 1 cm between segments. 1 cm cut-off has been shown to be consistent with the change in transection point when using fluorescence angiography.^11,12^ Randomly ordered letters were attributed to these segments to allow unbiased selection.

In total, 15 images were included, corresponding to 13 different patients. 2 patients had the same view pre- and post-fluorescence angiography (cases #8 and #10; and cases #9 and #14 respectively, supplementary figure 1), allowing a direct comparison of choices made on the same specimen.

### Participants

An online image bank was created. Participants were sent an invitation to join the study through the membership of the European Fluorescence Imaging-Guided Surgery (EURO-FIGS) registry. Each user was required to state their surgical and FA experience - previous experience with fluorescence (novice if under 5 uses of fluorescence angiography, expert otherwise); level of training (trainee or consultant); and area of specialisation (colorectal or not colorectal). The question “Would you use an automated system to standardise the decision of the transection point?” was completed by the study participants.

### Data Analysis

Users’ responses were analysed and compared by case, experience, level of seniority, and area of surgical specialization. The areas selected for transection were analysed as categorical variables using a one-way ANOVA test. A *P*-value under .05 was considered significant. Interrater agreement was assessed between raters for the different images.^13^ The Fleiss’ kappa statistic was calculated for every image.^14^ For comparison, 95% confidence intervals were obtained by bootstrapping using 100 repetitions with replacement. Stata (StataCorp., Texas, USA) version 14.1 was used for statistical analysis. Raters were excluded for agreement analysis if not all selections were entered.

### Ethics

All patients provided written informed consent for video data collection during surgery according to institutional guidelines and no patient identifiable data was used for the present study. No identifiable data was collected about participants in the survey and therefore no ethical approval was necessary.

## Results

The study included 108 participants. 7 participants were excluded from agreement analysis due to incomplete ratings on all images. The details of the participants are included in Table 1.

**Table 1.** Details of Study Participants.

Level of Training	% (n)
Consultants	64.8% (70)
Trainees	33.3% (36)
Missing data	1.9% (2)
Fluorescence experience	
Novice	42.6% (46)
Expert	56.5% (61)
Missing data	.9% (1)
Area of specialisation	
Colorectal	79.6% (86)
Not colorectal	19.4% (21)
Missing data	.9% (1)
Exclusions	6.5% (7)

Overall, there was no significant difference in the point of transection between any of the groups – consultant vs trainees; novice vs expert; or colorectal vs general – Table 2.

**Table 2.** Differences in Mean Normalised Score Between Different Rater Cohorts.

	# Ratings/Group	Mean Normalised Score	*P*-Value
Level of training			
Consultants	903	.622	.37
Trainees	454	.634	
Fluorescence experience			
novice	597	.626	.98
Expert	786	.627	
Area of specialisation			
Colorectal	1115	.627	.98
Not colorectal	255	.628	

The participant scores for each individual case and statistical analysis can be found in supplementary table 1.

Within each cohort, there was a poor level of agreement in responses. This was consistent throughout the different cohort categories as can be seen in Table 3.

**Table 3.** Agreement Analysis.

	# Raters	Agreement	95% CI
		.229	.184 - .287
Level of training			
Consultants	66	.24	.169 - .342
Trainees	32	.205	.153 - .262
Fluorescence experience			
novice	43	.206	.154 - .260
Expert	57	.251	.198 - .308
Area of specialisation			
Colorectal	80	.224	.170 - .300
Not colorectal	19	.258	.173 - .337

Of all participants, 68.5% replied yes to the question “Would you use an automated system to standardise the decision of the transection point?”.

## Discussion and Conclusions

The results of the present study show that there was no significant difference between the users when choosing a point of optimal bowel transection based on fluorescence angiography. There was no difference between consultants and trainees, fluorescence novices and experts, or colorectal subspecialists within colorectal surgery and generalists. Furthermore, there was a poor level of agreement within each cohort. These results reflect that the interpretation of fluorescence angiography images is not associated with expertise, seniority, or sub-specialism.

There is increasing evidence to suggest fluorescence angiography has a beneficial effect in reducing the risk of anastomotic leak in colorectal surgery. There have been a number of review articles recently^15,16^ which have shown this to be the case despite the lack of a large scale, formal randomised controlled trial. However, as with any technology or new technique it is important to be able to safely deploy so that the benefits may be obtained without any concerns over safety or complications. In the case of fluorescence angiography, the technique may be simple however any associated complications would be associated with misinterpretation of the images and thus sub-optimal clinical decision making. This is a novel situation for surgical technologies and is perhaps more attuned with radiology training which is determined by image interpretation. An example would be examining an x-ray and being able to interpret a fracture from normal bone based on the image characteristics.

Intuitively, one would have expected there to have been a difference between senior surgeons and trainees in determining the optimal transection point but that has not been the case in this study. That may be explained by the fact that optimal transection point could be argued to be a function of perfused bowel and maximal colonic length rather than just perfusion. It may be that the more junior surgeons have been more cautious whereas the more senior surgeons are attempting to maximise colonic length as well as perfusion.

A similar study by Nial et al.^9^ has also shown the variability amongst users of fluorescence angiography. In their study, video footage was used rather than still images with the raters being asked to position the optimal transection point rather than choosing from pre-selected options. Despite the slightly different methodology the results of the study were similar. This suggests that there is a need to incorporate formal image interpretation training as part of the implementation of fluorescence angiography for new users. Developing such a program would further augment the user’s ability to deploy fluorescence angiography in a more effective manner.

This study has certain limitations. The first is that we used still images rather than video. This means that the rater is unable to appreciate the dynamic flow of ICG into the tissue which could perhaps influence their decision-making. Using still images is possible as we had chosen frames which showed maximal peak intensity. The second limitation is the quality of the images themselves. As is the case in fluorescence angiography, the intensity of the fluorescence is not always reflective of perfusion, and this can be difficult to judge in such a survey. The rater may feel that the bowel is not adequately perfused when indeed this is the maximal peak intensity for that case. The third limitation is the rather rudimentary and arbitrary categorisation of experts, trainees, specialists, and generalists. There is currently no universal definition for this. Understanding what makes a user and expert is part of the challenge in fluorescence angiography.

In conclusion, the interpretation of fluorescence angiography images remains variable. There is no validated oral consensus agreement on the number of cases required to reach competency or expertise in interpretation. We suggest that in addition to training in the actual technique of administering ICG, there should be image interpretation training in parallel.

## Supplemental Material

Supplemental Material - Interobserver Variability in the Assessment of Fluorescence Angiography in the Colon Supplemental Material for Interobserver Variability in the Assessment of Fluorescence Angiography in the Colon by Antonio S. Soares, Neil T. Clancy, Sophia Bano, Imran Raza, Michelle Diana, Laurence B. Lovat, Danail Stoyanov, and Manish Chand in Surgical Innovation
