# Supplementary material for: Interobserver Variability in the Assessment of Fluorescence Angiography in the Colon
Source: Surg Innov. 2022 Nov 14;30(1):45–9. doi: 10.1177/15533506221132681 (PMC12174605; doi:10.1177/15533506221132681)
Supplement: Supplemental Material - Interobserver Variability in the Assessment of Fluorescence Angiography in the Colon [file sj-pdf-1-sri-10.1177_15533506221132681.pdf]

Supplementary table 1 - Differences in areas selected for transection

	**Mean scores**	**Difference in means**	**p-value**
**case #1**			
Novice	4.20	0.40	0.0331
Expert	3.80		
Trainee	4.10	0.20	0.4124
Consultant	3.90		
Colorectal	4.00	0.10	0.4636
Non-colorectal	3.90		
**case #2**			
Novice	3.02	-0.41	0.0063
Expert	3.43		
Trainee	3.11	-0.18	0.2721
Consultant	3.29		
Colorectal	3.33	0.13	0.4637
Non-colorectal	3.20		
**case #3**			
Novice	1.54	0.24	0.0193
Expert	1.30		
Trainee	1.55	0.20	0.0887
Consultant	1.35		
Colorectal	1.43	0.01	0.9428
Non-colorectal	1.42		
**case #4**			
Novice	4.87	-0.24	0.0482
Expert	5.11		
Trainee	4.94	-0.06	0.6843
Consultant	5.00		
Colorectal	5.05	0.08	0.6091
Non-colorectal	4.97		
**case #5**			
Novice	3.70	-0.20	0.3161
Expert	3.90		
Trainee	3.64	-0.20	0.3477
Consultant	3.84		
Colorectal	3.90	0.14	0.5645
Non-colorectal	3.76		
**case #6**			
Novice	3.52	0.18	0.3039
Expert	3.34		
Trainee	3.38	-0.02	0.953
Consultant	3.40		
Colorectal	3.05	-0.46	0.0296
Non-colorectal	3.51		
**case #7**			
Novice	4.37	-0.06	7.36E-01

Expert	4.43		
Trainee	4.36	-0.01	0.9551
Consultant	4.37		
Colorectal	4.38	0.00	
Non-colorectal	4.38		
**case #11**			
Novice	8.01	-0.12	0.8643
Expert	8.13		
Trainee	8.33	0.34	0.5319
Consultant	7.99		
Colorectal	7.90	-0.22	0.7432
Non-colorectal	8.12		
**case #12**			
Novice	1.83	0.15	0.2738
Expert	1.68		
Trainee	1.81	0.10	0.4882
Consultant	1.71		
Colorectal	1.75	0.01	0.972
Non-colorectal	1.74		
**case #13**			
Novice	6.00	-0.32	0.0565
Expert	6.32		
Trainee	6.11	-0.16	0.7287
Consultant	6.27		
Colorectal	6.15	0.00	0.9957
Non-colorectal	6.15		
**case #15**			
Novice	6.17	0.00	0.982
Expert	6.17		
Trainee	6.33	0.20	0.5535
Consultant	6.13		
Colorectal	6.25	0.01	0.9882
Non-colorectal	6.24		

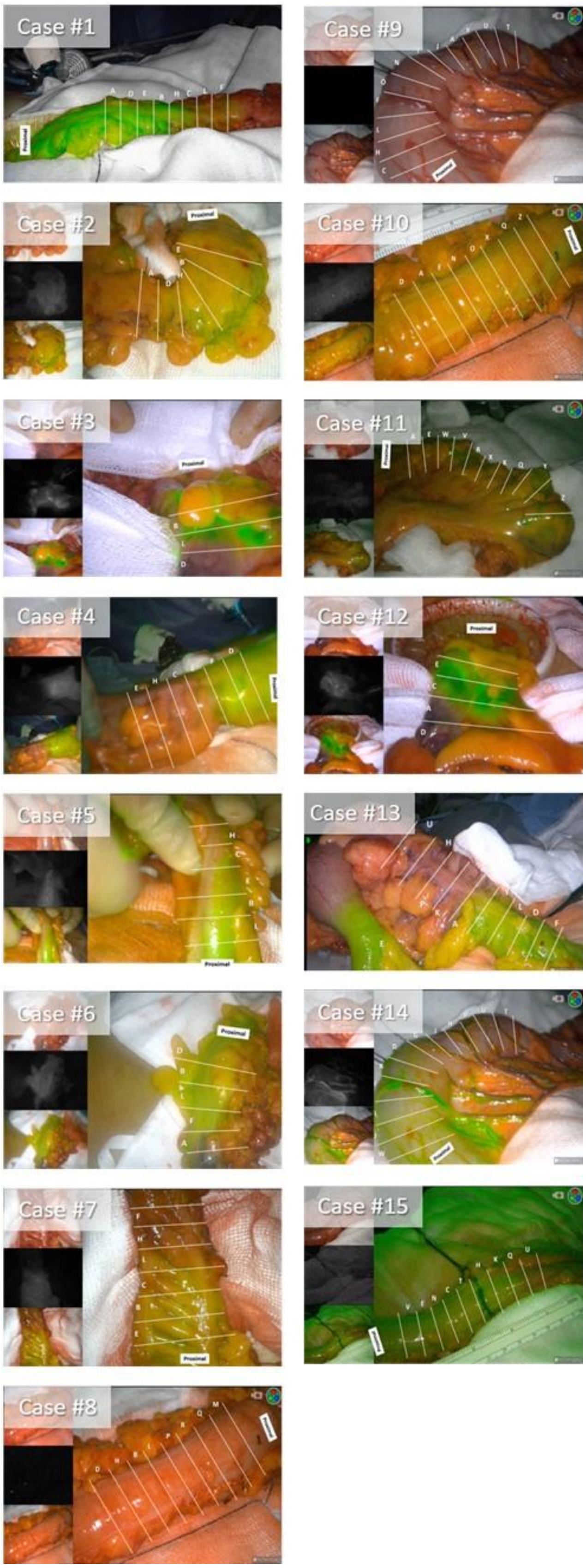

Supplementary Figure 1
